# Speech rehabilitation in children with cochlear implants using a multisensory (French Cued Speech) or a hearing-focused (Auditory Verbal Therapy) approach

**DOI:** 10.3389/fnhum.2023.1152516

**Published:** 2023-05-12

**Authors:** Lucie Van Bogaert, Laura Machart, Silvain Gerber, Hélène Lœvenbruck, Anne Vilain, Consortium EULALIES, Maud Costa

**Affiliations:** Université Grenoble Alpes, France; CRTLA, Centre Hospitalier Universitaire Grenoble Alpes, France; University of Alberta, Edmonton, Canada; Université Grenoble Alpes, France and Université de Montréal, Montréal, Canada; Université Grenoble Alpes, France; Memorial University, Newfoundland, Canada; ^1^Université Grenoble Alpes, Université Savoie Mont Blanc, CNRS, LPNC, Grenoble, France; ^2^Université Grenoble Alpes, CNRS, Grenoble INP, GIPSA-lab, Grenoble, France

**Keywords:** speech perception, children, hearing impairment, cochlear implant, Auditory Verbal Therapy, French Cued Speech, speech rehabilitation

## Abstract

**Introduction:**

Early exposure to a rich linguistic environment is essential as soon as the diagnosis of deafness is made. Cochlear implantation (CI) allows children to have access to speech perception in their early years. However, it provides only partial acoustic information, which can lead to difficulties in perceiving some phonetic contrasts. This study investigates the contribution of two spoken speech and language rehabilitation approaches to speech perception in children with CI using a lexicality judgment task from the EULALIES battery. Auditory Verbal Therapy (AVT) is an early intervention program that relies on auditory learning to enhance hearing skills in deaf children with CI. French Cued Speech, also called Cued French (CF), is a multisensory communication tool that disambiguates lip reading by adding a manual gesture.

**Methods:**

In this study, 124 children aged from 60 to 140 months were included: 90 children with typical hearing skills (TH), 9 deaf children with CI who had participated in an AVT program (AVT), 6 deaf children with CI with high Cued French reading skills (CF+), and 19 deaf children with CI with low Cued French reading skills (CF-). Speech perception was assessed using sensitivity (*d’*) using both the hit and false alarm rates, as defined in signal-detection theory.

**Results:**

The results show that children with cochlear implants from the CF- and CF+ groups have significantly lower performance compared to children with typical hearing (TH) (*p* < 0.001 and *p* = 0.033, respectively). Additionally, children in the AVT group also tended to have lower scores compared to TH children (*p* = 0.07). However, exposition to AVT and CF seems to improve speech perception. The scores of the children in the AVT and CF+ groups are closer to typical scores than those of children in the CF- group, as evidenced by a distance measure.

**Discussion:**

Overall, the findings of this study provide evidence for the effectiveness of these two speech and language rehabilitation approaches, and highlight the importance of using a specific approach in addition to a cochlear implant to improve speech perception in children with cochlear implants.

## 1. Introduction

The estimated incidence of childhood deafness before the age of two is two per 1,000 births (HAS, 2009). The developmental trajectories of deaf children are varied and different communication modalities are adopted. Some deaf children may use spoken language as their primary mode of communication, while others may use sign language, which is fully accessible to deaf people in terms of perception and production, as it does not rely on auditory perception. Some deaf children may also use a combination of both, known as bilingual and bimodal communication (sign language and spoken language). The choice between different interventions will depend on medical variables related to the child (etiology and severity of the deafness, associated comorbidities such as neurological disabilities) but also on the hearing status of the parents. Indeed, according to Hall et al. (2019), up to 95% of deaf children are born into homes where only spoken languages are in use. Thus, the parents’ own communication mode also influences their choice of communication modality for their child. In France, most parents (72% according to the survey in CISIC, 2021) opt for spoken communication with their child (CISIC, 2021) and cochlear implantation is a common practice (Parent et al., 2020). Yet data on spoken language skills in children with cochlear implants who are raised in a spoken communication environment are very scarce. The aim of the present study is to document the spoken language perception abilities in this population.

Many factors have been shown to influence speech perception in children with hearing impairment. Early detection of deafness, early intervention, and early hearing technology fitting are key factors in receptive language skills (O’Donoghue et al., 2000; Bubbico et al., 2007). The introduction of universal newborn hearing screening in many countries allows early detection of hearing loss and enables parents who opt for spoken communication to make early decisions about hearing aids, cochlear implantation as well as speech and language rehabilitation tools to be used at home.

While a hearing aid amplifies sound acoustically, a cochlear implant is a hearing device that requires an invasive medical surgery. It works by direct electrical stimulation to the auditory nerve. It is now a widely used hearing technology for profound deafness (when the inner part of the cochlea is damaged). There is an accepted view that cochlear implants improve speech perception (Gaylor et al., 2013; Kelsall et al., 2021). Indeed, it has been shown that restoration of sensory function by cochlear implants can reverse or reorganize some of the widespread neurocognitive effects of sensory loss (Kral et al., 2016). After cochlear implantation, many deaf children develop speech skills approaching those of their hearing peers, but heterogeneity is considerable (O’Donoghue et al., 2000; Geers et al., 2016). In congenitally deaf children, age at implantation is one of the main factors in the variability of outcomes (Boons et al., 2012). Indeed, studies have shown that early implantation before the age of 24 months improves on the development of phonological skills of children with cochlear implants (Colin et al., 2017). However, cochlear implantation is not always sufficient for a child to develop adequate hearing skills. One reason for this is that the acoustic signal provided by the implant remains degraded (Bouton et al., 2012). The implant provides only partial acoustic information, which can lead to difficulties in perceiving some phonetic contrasts. Limited phonological skills may in turn impact on spoken language development (Leybaert and LaSasso, 2010; Bouton et al., 2015; Hansson et al., 2018). In acoustically-degraded situations, lipreading has been shown to improve auditory speech perception (Sumby and Pollack, 1954). However, lipreading alone is not sufficient for deaf children to develop perfect speech perception. Out of context, lipreading only gives access to 10 to 30% of a word or a sentence (Bernstein et al., 2000). Thus, the use of a complementary communication tool is usually recommended to families and professionals to sustain the development of speech perception.

In this context, Cued Speech (Cornett, 1967) is one of the speech and language rehabilitation tools recommended by professionals in France. The theoretical underpinnings of this method are provided by a large bulk of studies describing speech perception as a multisensory process involving the integration of auditory and visual cues. Cued Speech is a manual code that complements the auditory-visual speech information in order to disambiguate visually identical phonemes and improve phonological decoding. A manual cue consists of two parameters: a handshape representing the consonant and a hand position around the face disambiguating the vowel (see Supplementary Appendix 1). A single manual cue can encode several phonemes, when these are distinguished by lip gestures (e.g., /m/, /t/ and /f/ are encoded with the same handshape but can be easily differentiated by lipreading). On the other hand, phonemes that share the same lip gesture are encoded with different manual cues (e.g., /p/, /b/ and /m/). Each language has its own Cued Speech, corresponding to its own phonological system. In this study, we investigated the benefits of French Cued Speech, also called Cued French (CF). It uses five hand positions around the face for vowels and eight handshapes for consonants. Cued Speech has been shown to improve speech perception in children with hearing impairment not only when audiovisual speech is presented together with manual cues (Ling and Clarke, 1975 for Cued Canadian English; Uchanski et al., 1994 for Cued American English; Périer et al., 1990 for CF) but also when cues are presented to children without auditory information (Nicholls and Ling, 1982). Moreover, a study on prelingually deaf French-communicating children reveals that the benefits of adding Cued Speech to lip-reading are higher when deaf children are exposed to Cued Speech early in life (Alegria et al., 1999). This study showed that CF substantially improved word and pseudo-word perception suggesting that CF corrects for lip-reading ambiguities. In addition, the improvement was more substantial in children exposed to CF at home before age 2 than in children exposed to CF later and solely at school. This suggests that the benefits of Cued Speech are most pronounced when it is introduced in early years and at home. It also implies that Cued Speech education is more beneficial when it is used in a more immersive way, rather than simply as part of a school curriculum. Furthermore, it has been suggested that Cued Speech exposure supports the development of more accurate phonological representations. Indeed, several studies showed that Cued Speech exposure leads to better phonological awareness, and promotes reading, spelling, and memorization (Bouton et al., 2011; Aparicio et al., 2012; Colin et al., 2017; Trezek, 2017). Even more strikingly, a study by Aparicio et al. (2012) revealed that Cued French education improves lipreading skills in adults, even when manual cues are absent, and Kos et al. (2009) demonstrated that it even improves audio speech perception, without any visual cue (Kos et al., 2009). More recently, Cued French reading skills has also been shown to improve speech production in children with cochlear implants (Machart, 2022).

In parallel to this multisensory tool, Auditory Verbal Therapy (AVT) is beginning to gain acceptance in France. AVT is a specialized and early intervention program for deaf children, that focuses on auditory perception training, with the premise that auditory learning is key to the development of listening, speaking and language skills (Eriks-Brophy, 2004). AVT is a family-centered coaching program. Parents are encouraged to use auditory verbal strategies in everyday life in order to stimulate their child’s auditory skills (Dettman and Dowell, 2010). Each AVT session includes activities designed to encourage development of the child’s listening skills, as well as ongoing assessment throughout the activities to adjust targets and provide continuous feedback to the child’s parents on progress. Regular assessments of listening, language, and speech skills are conducted using observation charts for younger children, or formal assessments with standardized tests for older children. Based on these assessments, specific goals are identified in collaboration with the family, taking into account the child’s age, cognitive abilities, and language and auditory skills. Additionally, four auditory activities are usually performed during the sessions: sound detection, sound discrimination, sound identification, and sound comprehension. For sound detection, the Ling’s Six Sounds Test (Ling, 1978) can be used, which involves identifying six sounds covering the frequency range of speech sounds ([a], [i], [u], [s], [∫], and [m]). This test helps determine whether the child can perceive all the sounds in the speech spectrum. The foundations of auditory-verbal practice are based on the 10 principles of the Alexander Graham Bell Academy for Listening and Spoken Language (see Supplementary Appendix 2). These principles include the promotion of early diagnosis of hearing loss, the use of appropriate hearing technologie, the parent coaching and the need to provide an environment that supports listening for the acquisition of spoken language throughout the child’s daily activities. While AVT is widely used and government-funded in some countries (e.g., Australia, Denmark, UK), there is still limited scientific evidence on its contribution to speech and language development. In a systematic review paper, Binos et al. (2021) selected and examined articles published in the last 10 years, that met specific quality criteria. Only eight papers were eligible. The authors concluded that although the reviewed papers showed a positive impact of AVT on speech and language skills of children with cochlear implants (CI), the lack of well-controlled studies and the small sample sizes makes it difficult to generalize. These studies did not include a control group of normal-hearing participants and most did not control for bias in participants’ age, age at implant or socioeconomic status (Binos et al., 2021). In one of these studies (Dettman et al., 2011), eight Australian children were enrolled in an AVT approach, 23 in an oralist approach (auditory-oral, AO) and 8 in bimodal bilingual communication (BB). The results showed that the AVT group performed better than the BB group in all measures of speech perception, but there was no significant difference between the AVT and AO groups. However, the hearing age in the AVT group was significantly higher than in the AO and BB groups. Another study compared the language skills of children with cochlear implants enrolled in different intervention approaches (Percy-Smith et al., 2018). The receptive and productive speech skills of 94 children who received “standard” Danish speech therapy were compared to those of 36 children who were enrolled in an AVT program. The “standard” rehabilitation consisted of speech and language therapy sessions once or twice a week and the therapy goals varied from child to child. Participants who received AVT had significantly higher scores in the three speech and language tasks, i.e., a receptive vocabulary test, a Danish test for active vocabulary and a spoken language comprehension test (Percy-Smith et al., 2018). Finally, Yanbay et al. (2014) compared the lexical performance, the auditory comprehension and the expressive skills of 42 children with cochlear implants enrolled in three communication programs: 18 in AVT, 12 in AO and 9 in BB. Results showed no difference between the three groups. Overall, there is some evidence that AVT can be beneficial for children’s speech and language development. However, the available studies are scant, have limitations and do not provide a clear consensus on the most effective approach. Additionally, the lack of a control group of typically-hearing children makes it difficult to determine whether the children in these studies are performing within the expected range for their age. A less recent review by Kaipa and Danser (2016) also shows that although some studies report that AVT can have a positive impact on spoken language skills, it is difficult to generalize their findings due to limited evidence and lack of well-controlled prospective studies. Future studies should use well-controlled group designs to minimize the role of external variables and strengthen the evidence for the benefits of AVT (Kaipa and Danser, 2016).

In the context of Evidence-Based Practice, clinicians are encouraged to make clinical decisions based on research evidence and national recommendations. Numerous approaches to sustain language and speech development in deaf children are used by clinicians. However, there is no consensus on the type of speech and language therapy that should be prioritized for deaf children (Gravel and O’Gara, 2003; Spencer et al., 2010; Demers and Bergeron, 2019). Several factors preclude drawing firm conclusions about the effectiveness of various intervention approaches. These include the lack of controlled studies, small sample sizes and the many variables that impact child development. National health authorities in France (“Haute Autorité de Santé,” HAS) recommend the use of tools involving multisensory modalities with deaf children (HAS, 2009). Therefore, HAS encourages the use of CF at home and at school. AVT, on the other hand, is not mentioned in the national guidelines.

Given the lack of scientific evidence to guide the choice of any particular method with deaf children, the preferred approach differs according to rehabilitation centers and countries. Some prefer a multisensory approach, arguing that speech perception is multisensory and that all phonemes should be made accessible and visible. Others favor an auditory-focused approach, arguing that the cerebral auditory regions need to be stimulated at an early age to develop adequate speech and language performance.

In this context, an exploratory survey was conducted to provide an overview of current speech and language intervention as well as educational practices with deaf children in France (Van Bogaert et al., 2021). The aim was to gain a clearer picture of the proportion of deafness professionals and parents currently using various language rehabilitation approaches with deaf children. The results obtained from 246 professionals and 215 parents showed that CF is used by 49% (*n* = 120) of professionals and 24% (*n* = 51) of parents. On the other hand, AVT is used by 1% (*n* = 3) of professionals and 8% (*n* = 23) of parents. Although AVT is rarely used, parents using this method reported being fully satisfied (by 98%) with it and stated that they observed language improvement in their child (by 97%) using this therapy. On the other hand, parents who engaged in CF at home were 79% satisfied and the percentage of progress observed by parents using CF was 72%. One explanation for this difference in satisfaction could be that parents using AVT at home chose this method freely, whereas parents using CF at home followed the national recommendations for the language development of the deaf child. In addition, the results of this survey reveal that AVT is introduced in France mainly through social networks and websites, unlike CF, which is implemented in families through professionals.

The co-existence of these two methods raises a fundamental scientific and therapeutic question, i.e., can multisensory and auditory-focused approaches be effective for the development of speech perception of deaf children with cochlear implants. In other words, can children who benefit from these approaches reach spoken language scores comparable to those of their peers with typical hearing? A complementary question is whether these two methods, which differ in the emphasis on the non-auditory modality contribute in a similar way to the improvement of spoken language skills.

The aims of this study are to determine whether both approaches allow the child to have speech perception within the expected range for chronological age and whether they differ in their contribution. The purpose is to better describe the current situation of children with CI in France, as concerns their phonological skills, according to the speech rehabilitation approach adopted by their families. This should lead to a better understanding of the strengths and weaknesses of these two methods and help clinicians to offer families different communication options, depending on the characteristics of the child and the family (Bergeron et al., 2020).

## 2. Materials and methods

The present study tested the performance of children with cochlear implants with different rehabilitation approaches to a lexicality judgment task, in comparison with a group of children with typical hearing.

The study was approved by the local ethics committee (CERGA, Comité d’Éthique pour les Recherches de Grenoble Alpes-Avis-2022-23). For all participants, written informed consent was obtained from the parents or caregivers and oral consent was obtained from all the children.

### 2.1. Participants

This study included 124 children (69 girls and 55 boys) aged between 60 and 140 months: 90 monolingual children with typical hearing skills (TH group) and 34 children with cochlear implants (CI group).

#### 2.1.1. Children with cochlear implants

For children with CI (see Table 1), inclusion criteria were (a) age between 5 and 11 years, (b) profound or severe hearing loss, (c) at least, one cochlear implant (d) native French speaker and (e) no diagnosed additional disorders. Apart from the child’s deafness, no additional disorders such as autism spectrum, behavioral disorders, learning disabilities, motor disorders, or intellectual deficits were diagnosed. However, all children were undergoing speech-language therapy, except for 7 children who were no longer in speech therapy. Table 1 shows the demographic characteristics of the 34 deaf children with cochlear implants.

**TABLE 1:** Socio-demographic information of children with cochlear implants.

	Chronological age (months)	Class	Gender	Deafness degree	First implant (months)	Unilateral or bilateral	Group
1	66	CP	M	Profound	17	Bilateral	AVT
2	81	CP	M	Profound	17	Bilateral	AVT
3	107	CE2	M	Profound	48	Bilateral	AVT
4	85	CE1	M	Profound	29	Bilateral	AVT
5	69	GSM	F	Profound	13	Bilateral	AVT
6	114	CM1	F	Profound	22	Bilateral	AVT
7	67	GSM	F	Profound	12	Bilateral	AVT
8	76	CP	M	Profound	11	Bilateral	AVT
9	85	CP	F	Profound	18	Bilateral	AVT
10	110	CE2	M	Profound	15	Bilateral	CF+
11	77	GSM	F	Profound	12	Bilateral	CF+
12	132	6e	F	Profound	18	Bilateral	CF+
13	91	CP	F	Profound	22	Bilateral	CF+
14	111	CM1	M	Profound	85	Bilateral	CF+
15	86	CP	M	Profound	20	Bilateral	CF+
16	100	CE1	M	Profound	36	Bilateral	CF−
17	103	CE2	F	Profound	55	Bilateral	CF−
18	114	CM1	F	Profound	12	Bilateral	CF−
19	139	6e	F	Profound	19	Bilateral	CF−
20	74	CP	M	Profound	28	Bilateral	CF−
21	96	CM1	M	Profound	90	Bilateral	CF−
22	135	CM2	M	Profound	18	Bilateral	CF−
23	74	CP	M	Profound	11	Bilateral	CF−
24	65	GSM	M	Profound	49	Unilateral	CF−
25	72	GSM	F	Profound	12	Bilateral	CF−
26	94	CE1	M	Profound/severe	60	Bilateral	CF−
27	111	CE2	M	Profound	26	Bilateral	CF−
28	64	MSM	F	Profound	15	Bilateral	CF−
29	99	CE2	F	Profound	22	Bilateral	CF−
30	120	CM1	F	Profound	16	Bilateral	CF−
31	77	CP	M	Profound	14	Bilateral	CF−
32	86	CE1	M	Profound	19	Bilateral	CF−
33	92	CE1	M	Profound	19	Bilateral	CF−
34	84	CP	M	Profound	23	Unilateral	CF−

GSM, Year 1, age 5 to 6 (Kindergarten); CP, Year 2, age 6 to 7 (1st grade); CE1, Year 3, age 7 to 8 (2nd grade); CE2, Year 4, age 8 to 9 (3rd grade); CM1, Year 5, age 9 to 10 (4th grade); CM2, Year 6, age 10 to 11 (5th grade); 6e, Year 7, age 11 to 12 (6th grade).

The CI group includes 34 children (14 girls and 20 boys) with profound hearing loss, with cochlear implants (chronological age = 92.82 months, SD = 20.86). Details are shown in Table 2. All of them have been exposed to French since birth and are native French speaker, with no daily exposure to another language. They all lived in France, but in different regions. Seven children were occasionally exposed to French Sign Language (FSL) at school or at home but their level of FSL was low, according to their parents. Only one child was judged to have a very good level of FSL by the parents. This child was removed from our sample to control for multilingualism. The other deaf children (*n* = 27) had never been exposed to FSL. All parents were typically hearing. Thirty-two children in the sample wore two implants (bilateral implantation) and two children had a single implant (unilateral implantation). The mean age at cochlear implantation for children with CI was 27.11 months (SD = 21.04).

**TABLE 2:** Chronological age and age at implantation in months [minimum-maximum (mean; sd)].

	*N*	Chronological age (months)	Age at first CI (months)
AVT	9	77–132 *(83.33; 17.09)*	12–85 *(20.78; 11.61)*
CF**+**	6	65–139 *(101.17; 20.21)*	11–90 *(28.67; 27.82)*
CF−	19	66–114 *(94.68; 21.97)*	11–48 *(29.63; 22.55)*
TH	90	61–131 *(84;69; 16.08)*	

This group was divided into three subgroups: AVT, CF+, and CF-. The first group (AVT) consists of 9 children with cochlear implants who had participated in an AVT program of at least 2 years. The second group (CF+) consists of 6 children with cochlear implants with high CF reading skills. The third group (CF-) consists of 19 deaf children with cochlear implants with low CF reading skills. CF reading proficiency was measured using the TERMO test (Busquet and Descourtieux, 2000).

The mean age at first cochlear implantation was 20.78 months (SD = 11.61) for the AVT group, 28.67 months (SD = 27.82) for the CF+ group and 29.63 months (SD = 22.55) for the CF- group (see Table 2).

According to the parents, the CI hearing thresholds of all children were in the range from 15 to 30 dB, except for one child whose CI hearing threshold was 40 dB. Unfortunately, we were unable to obtain CI hearing thresholds for three children because the parents did not know them. The mean CI hearing thresholds was 24.5 dB (±4.17) for the AVT group, 22.5 dB (±2.88) for the CF+ group and 25 dB (±6.27) for the CF- group. It should be noted that hearing is classified as normal when the hearing threshold falls within the range of 0–20 dB, while a hearing loss is considered as mild when the threshold falls between 20 and 40 dB.

For children exposed to Cued French, we collected information on the frequency of Cued French use (daily/weekly/occasional), the earliness of exposure to cued speech (since birth/since kindergarten/since primary school) and the places where Cued French was used (home/school). Table 3 provides the number of children in each category.

**TABLE 3:** Information about the frequency of Cued French use, the earliness of exposure to Cued French and the places of Cued French use.

	Frequency of Cued French use			Earliness of exposure to Cued French			Places of Cued French use		
	**Daily** ***N* = 9**	**Weekly** ***N* = 9**	**Occasional** ***N* = 6**	**Birth** ***N* = 7**	**Kindergarten** ***N* = 12**	**Primary school** ***N* = 5**	**Home/School** ***N* = 14**	**Home** ***N* = 6**	**School** ***N* = 4**
CF+	N = 5	*N* = 1	*N* = 0	*N* = 4	*N* = 1	*N* = 1	*N* = 5	*N* = 1	*N* = 0
CF−	*N* = 4	*N* = 8	*N* = 6	*N* = 3	*N* = 11	*N* = 4	*N* = 9	*N* = 5	*N* = 4

The parents of children in the AVT group were asked about the age their child had started and finished therapy, and the average program duration was calculated. The average duration of therapy for the children in the AVT group was 39.22 months, with a range of 24–61 months.

The AVT children were recruited through the association ADEFAV (Association des Familles AVTistes)^1^ which promotes language rehabilitation through hearing. The children exposed to cued speech were recruited during the ALPC summer camp (Association Nationale pour la Langue Parlée Complétée)^2^ or through the Grenoble University Hospital.

#### 2.1.2. Children with typical hearing skills

The 90 TH children (55 girls and 35 boys) belonged to the large cohort of typical children in the EULALIES project (Meloni et al., 2017). All TH children had working memory (assessed with the verbal digit span) and morphosyntactic skills (assessed with the ELO sentence production task, cf. infra) within the expected range for their age. The mean chronological age of this group was 84.69 months (SD = 16.08). None of the children were daily exposed to another language than French. All TH children were recruited from schools in the Grenoble area.

### 2.2. Procedure and experimental tasks

The experiment took place in a quiet room. The child sat at a table in front of a computer screen. A loud speaker connected to the computer was placed in front of the child and the sound level was set at 80 dB for optimal listening conditions. This set-up was used to simulate a listening situation during a typical one-to-one interaction. The child wore a head-mounted microphone (Shure) which was connected to a Zoom H4N Pro digital audio-recorder, to record his/her speech production. The experimenter sat to the child’s right, facing the computer screen. On average, the assessment procedure took 45 min to complete. For children with typical hearing skills, a pure-tone audiometric screening was conducted to eliminate a possible hearing disorder (perception at 20 dB on the frequencies 250 Hz, 500 Hz, 1,000 Hz, 2,000 Hz, 4,000 Hz, and 8,000 Hz).

The experiment began with a digit span task from the ODEDYS battery (Outil de DEpistage des DYSlexies, Jacquier-Roux et al., 2005). The aim of this task was to assess short term verbal working memory.

Then, the child performed three speech production and perception tasks from the EULALIES battery (Meloni, 2022): picture naming, lexicality judgment, and non-word repetition. This battery enables to collect data on speech production and perception in typically- and atypically-developing children and to provide standardized data on speech sound development in French. The picture-naming task assesses the accuracy of spontaneous phoneme production in isolated familiar word context. It allows the analysis of phonological production skills, using all the phonemes of French. The lexicality judgment task is designed to analyze the child’s perceptual judgment. The non-word repetition task consists in asking the child to repeat unknown items presented audio-visually (sound + lip gestures).

In this paper, we focus on the lexicality judgment task from the EULALIES battery, which assesses phonological perception. In this task, the child sees a picture corresponding to a familiar item and then watches a video of a speaker naming the item (sound + face of the speaker). The child has to judge whether the French word is correctly pronounced or whether there is a phonological alteration in the word (e.g., indicating whether [pomat} corresponds to the picture of a tomato, [tomat] in French). All items are illustrated by a picture and are the same frequent items as in the picture naming task, which is performed just before the lexicality judgment task. This is meant to avoid a bias in vocabulary knowledge. The task consists of 90 items: 45 non-phonologically altered items and 45 items with phonological alteration. The alterations are present at the level of word structure (metathesis, cluster simplification, syllable deletion, segment deletion, and segment inversion) or at the segmental level (phoneme substitutions). The alterations occur on the initial, medial, or final position of the word and the number of syllables per item varies from 1 to 4 syllables. The stimuli in this task were carefully designed to contain phonological alternations that are often observed in speech sound disorders and which may reflect phonological awareness deficits (Meloni, 2022). Table 4 describes the altered words used in the task and the corresponding types of alterations (see Supplementary Appendix 3 for the number of items within each category of alteration). Five lists were designed in which the items were presented in a different and random order to limit possible biases related to a succession of alterations.

**TABLE 4:** Summary of the phonologically altered stimuli used in the lexicality judgment task.

	Correct stimuli	Incorrect stimuli	Translation	Alteration on the segment or structure	Type of alteration	Number of features substituted	Position of alteration	Number of syllables
1	Gare [gaʁ]	[ga∫]	Station	Segment: consonant	Substitution: place (fronting) and devoicing	2	Final	1
2	Tigre [tigʁ]	[ti]	Tiger	Structure	Cluster deletion		Final	1
3	Zèbre [zεbʁ]	[zεʁb]	Zebra	Structure	Metathesis		Final	1
4	Enveloppe [ɑ̃vlɔp]	[ɑ̃vlɔt]	Envelope	Segment: consonant	Substitution: place (backing)	1	Final	2
5	Locomotive [lokomotiv]	[jokomotiv]	Locomotive	Segment: consonant	Substitution: place (backing), manner (gliding)	2	Initial	4
6	Fourchette [fuʁ∫εt]	[fyʁ∫εt]	Fork	Segment: vowel	Substitution: place	1	Medial	2
7	Déguisement [degizmɑ̃]	[tegizmɑ̃]	Fancy dress	Segment: consonant	Substitution: devoicing	1	Initial	3
8	Farine [faʁin]	[baʁin]	Flour	Segment: consonant	Substitution: manner (stopping), place (fronting) and voicing	3	Initial	2
9	Neige [nɛʒ]	[nɛʃ]	Snow	Segment: consonant	Substitution: devoicing	1	Final	1
10	Docteur [dɔktœʁ]	[sɔktœʁ]	Doctor	Segment: consonant	Substitution: manner (frication) and devoicing	2	Initial	2
11	Griffe [ɡʁif]	[ɡʁiz]	Claw	Segment: consonant	Substitution: place (backing) and voicing	2	Final	1
12	Camion [kamjɔ̃]	[kanjɔ̃]	Truck/lorry	Segment: consonant	Substitution: place (backing)	1	Medial	2
13	Hippopotame [ipopotam]	[ipopopam]	Hippopotamus	Segment: consonant	Substitution: place harmonization (backing)	1	Medial	4
14	Ciseaux [sizo]	[sezo]	Scissors	Segment: vowel	Substitution: aperture	1	Medial	2
15	Aquarium [akwaʁjɔm]	[akwajɔm]	Aquarium	Structure	Phoneme deletion		Medial	3
16	Avion [avjɔ̃]	[apjɔ̃]	Airplane	Segment: consonant	Substitution: manner (stopping), place (fronting) and devoicing	3	Medial	2
17	Main [mmɛ̃]	[mε]	Hand	Segment: vowel	Substitution: denasalization	1	Final	1
18	Couverture [kuvɛʁtyʁ]	[kuvɛʁtyk]	Blanket	Segment: consonant	Substitution: place (fronting), manner (stopping) and devoicing	3	Final	3
19	Livre [livʁ]	[liʁv]	Book	Structure	Metathesis		Final	1
20	Citron [sitʁɔ̃]	[sitʁɑ̃]	Lemon	Segment: vowel	Substitution: aperture	1	Final	2
21	Cinéma [sinema]	[tinema]	Cinema	Segment: consonant	Substitution: manner (stopping)	1	Initial	3
22	Bonhomme [bonɔm]	[bojɔm]	Man	Segment: consonant	Substitution: manner (gliding), place (backing) and denasalization	3	Medial	2
23	Telephone [telefɔn]	[telefɔv]	Phone	Segment: consonant	Substitution: place (fronting), denasalization and manner (frication)	3	Final	3
24	Poisson [pwasɔ̃]	[pwaʃɔ̃]	Fish	Segment: consonant	Substitution: place (backing)	1	Medial	2
25	Toboggan [tobogɡɑ̃]	[togobɡɑ̃]	Slide	Structure	Metathesis		Medial	3
26	Grenouille [ɡʁənuj]	[ʁənuj]	Frog	Structure	Phoneme deletion (cluster simplification)		Initial	2
27	Biberon [bibʁɔ̃]	[blibʁɔ̃]	Baby bottle	Structure	Phoneme insertion (cluster insertion)		Initial	2
28	Pieuvre [pjœvʁ]	[pjœʁv]	Octopus	Structure	Metathesis		Final	1
29	Chaussette [∫osεt]	[sosεt]	Sock	Segment: consonant	Substitution: place harmonization (fronting)	1	Initial	2
30	Capuche [kapy∫]	[kapys]	Hood	Segment: consonant	Substitution: place (fronting)	1	Final	2
31	Loup [lu]	[lɔ̃]	Wolf	Segment: vowel	Substitution: aperture and nasalization	2	Final	1
32	Chocolat [∫okola]	[ʃokʁola]	Chocolate	Structure	Phoneme insertion (cluster insertion)		Medial	3
33	Voiture [vwatyʁ]	[tyʁ]	Car	Structure	Syllable deletion		Initial	2
34	Tomate [tomat]	[pomat]	Tomato	Segment: consonant	Substitution: place (fronting)	1	Initial	2
35	Menton [mɑ̃tɔ̃]	[mɔ̃tɔ̃]	Chin	Segment: consonant	Substitution: aperture harmonization	1	Medial	2
36	Éléphant [elefɑ̃]	[lefɑ̃]	Elephant	Structure	Syllable deletion		Initial	3
37	Crocodile [kʁokodil]	[kʁokʁodil]	Crocodile	Structure	Phoneme insertion (cluster harmonization)		Medial	3
38	Dentiste [dɑ̃tist]	[dɔ̃tist]	Dentist	Segment: vowel	Substitution: aperture	1	Medial	2
39	Œuf [œf]	[ɔf]	Egg	Segment: vowel	Substitution: place	1	Initial	1
40	Medicament [medikamɑ̃]	[dikamɑ̃]	Medication	Structure	Syllable deletion		Initial	4
41	Aspirateur [aspiʁatœʁ]	[ɔspiʁatœʁ]	Vacuum cleaner	Segment: vowel	Substitution: aperture and place	2	Initial	4
42	Uniforme [ynifɔʁm]	[ynifɔʁp]	Uniform	Segment: consonant	Substitution: denasalization and devoicing	2	Final	3
43	Hibou [ibu]	[ibo]	Owl	Segment: vowel	Substitution: aperture	1	Final	2
44	Bibliothèque [biblijotεk]	[bliblijotεk]	Library	Structure	Phoneme insertion (cluster harmonization)		Initial	4
45	Escargot [εskaʁgo]	[εksaʁgo]	Snail	Structure	Metathesis		Medial	3

At the end of these tasks, the morphosyntactic level was assessed using the sentence production task from the ELO battery (Khomsi, 2001). It is part of a battery designed to describe and assess children’s oral language in reception and production, and to identify language disorders. To complete this task, children have to produce morphosyntactic features, by completing 25 sentences.

For children exposed to Cued French (CF), the level of CF reading skills was assessed using the *“Test d’évaluation de la réception du message oral par l’enfant sourd”* (TERMO, Evaluation test for the reception of the oral message by the deaf child; Busquet and Descourtieux, 2000). It consists in presenting children with words and sentences in visual form alone (lip gestures + manual cues) without sound. The child is asked to repeat the items vocally. We assessed only lexical accuracy (the phonological aspect of their production was not assessed). Two levels of CF were determined: low CF reading skills (CF-) and high CF reading skills (CF+). Children in the CF- group could at most understand a few familiar words at normal speech rate (levels 3 and 4 of the TERMO scale). Children in the CF+ group could comprehend familiar words and sentences at normal speech rate (levels 1 and 2 of the TERMO scale).

Finally, the parents were asked to complete a questionnaire, in order to collect socio-demographic information about each child. Parents were asked to provide information about their occupation, the child’s school level, information about their child’s hearing impairment (degree of deafness, age of screening, hearing threshold, type of hearing aid, date of hearing aid fitting, frequency of use of the hearing aid). Information on the mode of communication used with the child (AVT, cued speech, LSF, or other) and languages spoken at home was collected. Additional questions provided more detail on the frequency of use (daily/weekly/occasional), earliness of use (since birth/since kindergarten/since primary school), and place of use (home/school/speech therapist) of the different modes of communication.

### 2.3. Data processing and statistical analyses

All graphs and statistical analyses were completed using the R software (R Core Team, 2006).

#### 2.3.1. Working memory and morphosyntax skills

The ODEDYS digit task (Jacquier-Roux et al., 2005) was used to measure working memory skills. To compare the working memory skills of our participants, we converted the scores of the participants in the task into a standard score (Z score) computed relative to a group of typical hearing children. This allows to correct for chronological age in the analyses. Regarding the morphosyntax skills, we used the morphosyntax production subtask of the ELO battery (Khomsi, 2001). We also used a standard score (Z score).

#### 2.3.2. Lexicality judgment task

For the lexicality judgment task, the correct acceptation score (coded 1 when the child had correctly identified that the phonological form of the word was correct, and 0 when s/he indicated that the phonological form of the word was incorrect) and the correct rejection score (coded 1 when the child correctly identified that the phonological form of the word was incorrect, and 0 when s/he didn’t correctly identify that the phonological form of the word was incorrect) were converted to *d’* scores, derived from signal detection theory. The *d’* score is a method that calculates the perceptual discrimination sensitivity, independently of the decision strategy in discrimination or categorization tasks (Macmillan and Creelman, 2004). These analyses are based on Hits (i.e., correct acceptation), False Alarms (i.e., errors on acceptation), Correct Rejection, and Miss (i.e., errors on rejection). This score was calculated using the *dprime* function of the R software (R Core Team, 2006). The research question aims to analyze the relationship between the individual *d’* scores (participant’s perceptual discrimination sensitivity) and the chronological age in different groups of children with cochlear implants (CI) compared with typically-hearing peers. Specifically, we are comparing the performance of a group of children with typical hearing (TH) with that of three groups of children with CI (i.e., AVT, CF+, CF-). For that purpose, we first assessed the relationship between the individual *d’* score and the chronological age (in months) of the TH participants. Since this relationship is not linear (*d’* values increase faster with chronological age at the beginning of the observation domain than at the end, and tends toward an asymptote), we computed an exponential type model with an asymptote using the *nls* function of the R software. We then constructed a 95% prediction interval for the estimated curve. This allowed us to situate the participants in the CI groups in relation to this interval and thus to know what proportion of these participants were outside or inside the TH prediction interval. To do this, we used the *predFit* function from the *investr* package of the R software. In addition, for each CI groups (i.e., AVT, CF+, CF-), we performed a proportion compliance test to assess whether the proportion of participants outside the TH prediction interval was significantly different from 5% (the expected proportion in the TH group). For this purpose, we used the *binom.test* function of the R software. Finally, for the children from the CF+, CF-, and AVT groups who were outside of the TH prediction interval, we calculated the difference between their *d’* scores and the lower boundary of the prediction interval at their age. We then averaged these differences for each group.

This correct-incorrect task is subject to response bias. In order to quantify this bias, the β score (also calculated from the *dprime* function of the R software) measures the participant’s strategy for performing the speech perception task (Macmillan and Creelman, 2004). Thus, it calculates how biased the participant is in the lexicality judgment task. A β score of 1 indicates that the participant’s behavior is unbiased. When the β score is between 0 and 1, it means that the behavior is liberal. Finally, a score of 1 to infinity on the β indicates that the behavior is conservative/cautious. The methodology used for the analysis of the β score is similar to that described above, with one exception. Indeed, the evolution between the individual β score and the chronological age (in months) appears to be linear, or even constant over the entire observation domain. We then chose to model this relationship with a linear regression using the *lm* function of the R software.

#### 2.3.3. Types of phonological alterations

To analyze the speech perception performance on specific types of phonological alterations, we classified the alterations into seven categories:

– Consonant substitution (when a consonant is replaced by another consonant).
– Harmonization (when a phoneme or a group of phoneme becomes similar to a neighboring phoneme).
– Metathesis (when two phonemes are inverted).
– Phoneme deletion (when a phoneme is deleted).
– Phoneme insertion (when a phoneme is added).
– Syllable deletion (when a syllable is deleted).
– Vowel substitution (when a vowel is replaced by another vowel).

This analysis was meant to determine whether children with cochlear implants (CI) in the three groups had difficulties with a particular type of phonological alteration, based on the speech rehabilitation approach used. We expected certain types of phonological alterations to be particularly challenging for CI children, and we suspected that there might be differences between the AVT and CF+ groups with regards to the different phonological alterations. Identifying the most difficult types of phonological alterations for CI children could allow clinicians to design specific interventions to enhance speech perception.

The statistical analysis was run using a linear model and pairwise comparisons with the *emmeans* function in R software, with group and type of alteration as fixed effects.

#### 2.3.4. Variables that influence Cued French reading skills

The two groups of CF children in this study are differentiated with their Cued French reading skills (cf. Section “2.2. Procedure and experimental tasks”). However, other factors might have explained their Cued French reading skills, such as language skills or verbal working memory. In order to assess the variables that explained the children’s cue reading skills, two types of statistical analyses were run. They tested whether the variables “frequency of Cued French use” (daily/weekly/occasional), “earliness of exposure to Cued French” (since birth/since kindergarten/since primary school), “place of Cued French use” (home/school/speech therapist), “morphosyntactic skills,” and “verbal working memory skills” have an impact on whether a child belongs to the CF+ or CF- group.

In the first method, we performed a logistic regression. In the initial model, we integrated all the five variables. Then, we used the Akaike Information Criterion (AIC) to remove variables that did not provide additional information, given the presence of the others, on the probability of belonging to the CF+ or CF- group. The AIC is a measure of the relative quality of a statistical model for a given set of data. It is based on the likelihood function, which quantifies the goodness of fit of a model to the data, and takes into account the complexity of the model. For this we used the *glm* function and the *step* function of the R software.

Random forest analysis (Breiman, 2001) was used in the second method. Random forests are a type of ensemble learning method that can be used for classification and regression tasks. For this, we used the *randomForest* function and the *varImPlot* function of the *randomForest* package of the R software. The *varImPlot* function is used to rank the most influential variables for the correct assignment of participants to the CF+ and CF- group. Random permutations are made for each variable and the extent to which this changes the outcome of the predictions is examined. The more influential a variable is, the greater the impact of permutations on the results.

## 3. Results

### 3.1. Working memory

Verbal working memory was analyzed using the standardized score (Z score) of the ODEDYS digit verbal span task.

**FIGURE 1:**
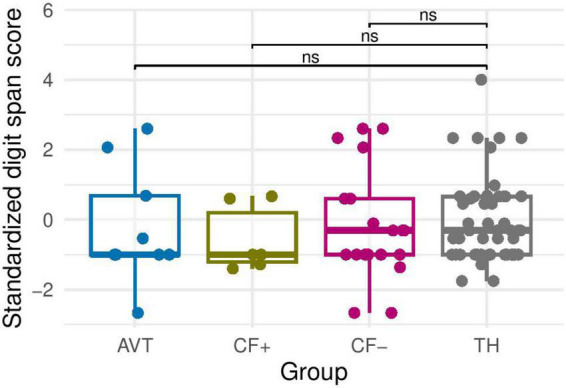
Verbal memory Z score (standardized digit span score) for the four groups (AVT, CF+, CF− and TH). ns, non-significant.

The normality assumption was checked by using the Shapiro-Wilk test. Results showed that the distribution of the data was significantly different from the normal distribution. In this context, we used the non-parametric Wilcoxon rank sum test to compare two independent sample groups. The Wilcoxon test showed no significant difference (*p* > 0.05) between the 4 groups. Results indicated that the working memory (measured by the verbal digit span task) did not differ between groups (see Figure 1).

### 3.2. Morphosyntax skills

**FIGURE 2:**
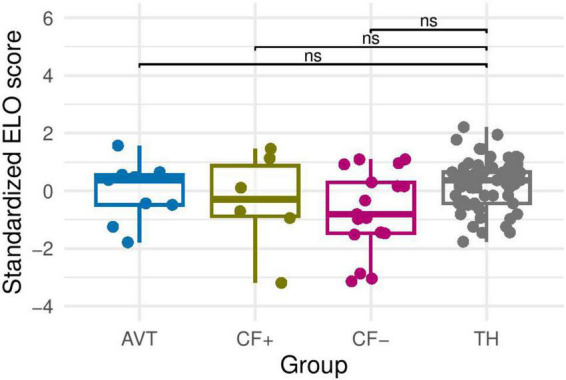
Morphosyntax Z score (standardized ELO score) for the four groups (AVT, CF+, CF− and TH). ns, non-significant.

Firstly, we checked the normality of the data. The Shapiro test showed that the data followed a normal distribution. However, the Levene test, being significant, showed that the homogeneity of variances was not verified. Secondly, we performed a Welch’s *t*-test, adapted for samples with unequal variances. The results showed no differences between the four groups (*p* > 0.05) (see Figure 2).

### 3.3. Age at cochlear implantation

**FIGURE 3:**
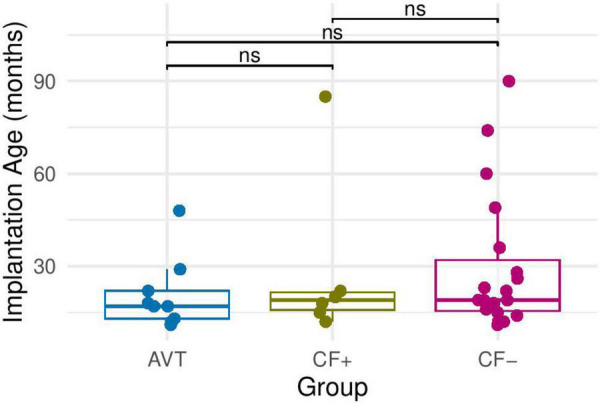
Implantation age in months in each of the CI groups: AVT, CF+, CF−. ns, non-significant.

To analyze whether or not participants in the CI groups differ in age at cochlear implantation, we also used the non-parametric Wilcoxon rank sum test because the normality of the distribution was not verified. The results show no differences between the AVT group, the CF+ group and the CF- group. This means that the three groups of children have equivalent age at cochlear implantation (see Figure 3).

### 3.4. Lexicality judgment

**FIGURE 4:**
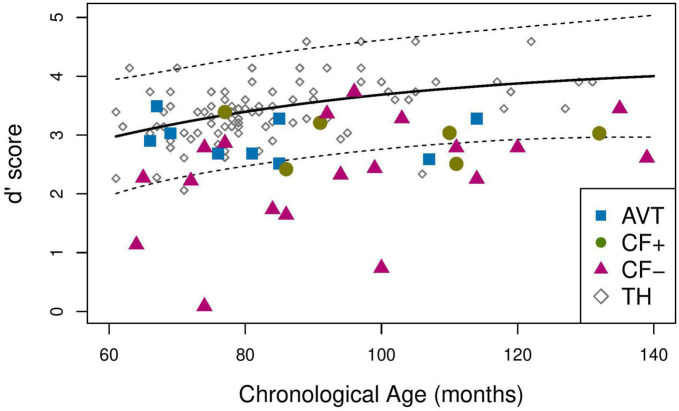
Mean *d*’ score for the TH (solid line), 95% prediction interval for the TH group (dotted line).

To analyze the performance of the children in the CI groups on the lexicality judgment task compared to TH children, perceptual discrimination sensitivity was first examined. The *d’* score for each participant was computed and plotted as a function of chronological age. As presented in Figure 4, in children with TH, the increase in *d’* score as a function of chronological age can be modeled using an exponential model with an asymptote (solid line). A linear regression analysis was used to test if age at cochlear implantation significantly predicted the *d’* score in the children with CI. The regression was not statistically significant (*p* > 0.05). This suggest that the age of cochlear implantation does not significantly influence the results and will not be considered in the following statistical analyses.

In order to assess whether the sensitivity of the children with CI in the three groups were similar to that of the children in the TH group, we plotted the 95% prediction interval for the *d’* scores of the children in the TH group (Figure 4, dotted lines). We then examined whether the *d’* scores of the children with CI belonged to this interval or how much below the interval their scores were. Table 5 provides the proportion of participants outside the TH 95% prediction interval (i.e., children whose *d’* value is below the lower bound of the interval), for the three CI groups. Table 5 also gives the mean distance between the *d’* score of each child whose score lies below the TH prediction interval, and the lower boundary score, for the three CI groups (i.e., AVT, CF+, and CF-).

**TABLE 5:** Number of children outside the TH 95% prediction interval and mean distance from the prediction interval for each group.

	AVT group	CF+ group	CF− group
Number of children outside the 95% TH prediction interval (n/N) of the *d’*score	2/9	2/6	12/19
Mean distance from the prediction interval	0.14	0.25	0.75

The proportion compliance test reveals that the proportion of participants in the CF- group below the prediction interval is significantly different from 5%, i.e., from the expected proportion in the TH group (*p* < 0.001). The same result is found for the CF+ group (*p* = 0.033). The participants in the AVT group also tend to have scores that differ from those of children with TH (*p* = 0.07). In sum, there is a significant probability for children with CI to have a lower *d’* score than a TH participant of the same age in the observation domain (60–140 months).

However, the distance analysis reveals that the distance to the TH group differs for the three groups of children with CI. For participants with CI outside the 95% prediction interval of the TH group, the average distance between the lower bound of this interval and the *d’* value is greatest for CF- participants (0.75), followed by CF+ participants (0.25), and AVT participants (0.14) (see Table 5).

**FIGURE 5:**
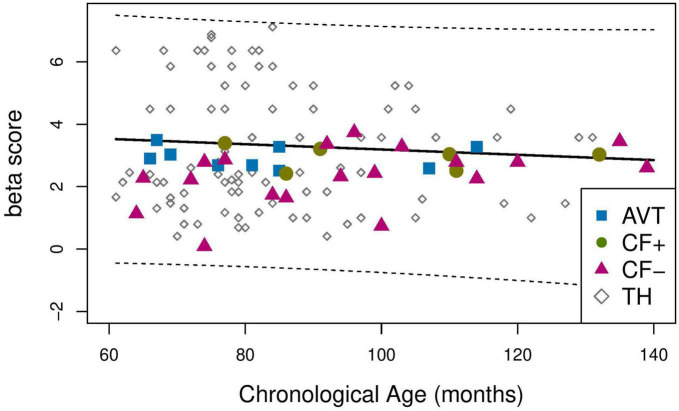
Mean *b* score for the TH (solid line), 95% prediction interval for the TH group (dotted line).

Secondly, we examined the perceptual bias, by computing β score and by running the same analysis as for the *d’*. None of the children with CI were outside the 95% prediction interval of the TH. The range of the prediction interval is very large because of the large variability of beta values for TH participants. This suggests that, at any given chronological age, the groups of CI children do not significantly differ from their TH peers in terms of β values (see Figure 5).

### 3.5. Type of phonological alteration

**FIGURE 6:**
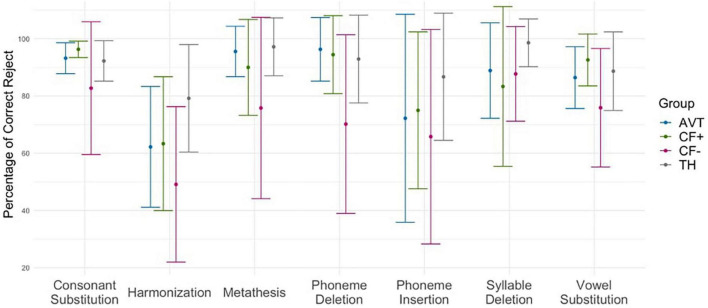
Percentage of correct rejections for each type of phonological alteration across the four groups (CF+, CF−, AVT, and TH).

The pairwise comparison analysis of correct rejection performance for each type of phonological alteration and each group showed that the CF- group had significantly lower performance in comparison with the TH group (*p* < 0.001), except for syllable deletion and phoneme insertion, for which there were no differences between groups. The CF- group also had significantly lower scores than the CF+ and AVT groups for consonant substitution and vowel substitution. The scores of the CF+ and AVT groups were not different from those of the TH group, except for a marginal difference between AVT and TH on harmonization (*p* = 0.09). The Figure 6 illustrates the percentage of correct rejections for each type of phonological alterations across the four groups.

### 3.6. Variables that influence Cued French reading skills

We used the AIC information criterion to select the variables that provide information on the probability of belonging to the CF+ or CF- group for the linear regression. The results show that the variables “morphosyntax score” and “verbal working memory score” do not provide any additional information when the other three variables (“frequency of Cued French use,” “earliness of Cued French exposure,” and “place of Cued French use”) are taken into consideration. The presence of the other three variables has an impact on the probability of belonging to the CF+ or CF- group, and the morphosyntax score and verbal working memory score do not provide any additional information in this regard.

**FIGURE 7:**
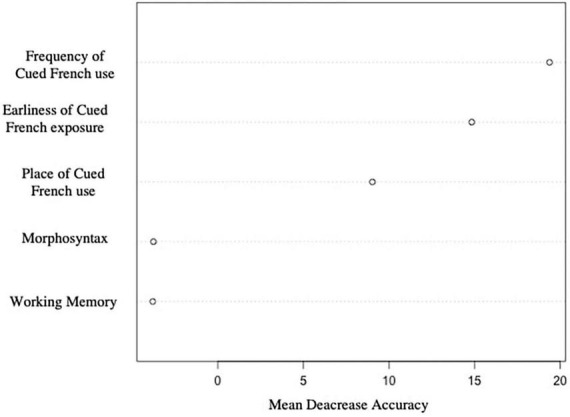
Relative influence of each variable on Cued French reading skills (a high mean decrease accuracy corresponds to a high role in the separation between CF+ and CF− groups).

The random forest method shows that the most influential variables for the correct assignment of participants to the CF+ and CF- groups are in order: “frequency of Cued French use”–“earliness of Cued French exposure”–“place of Cued French use”–“verbal working memory score”–“morphosyntax score.” More precisely, Figure 7 shows the influence of the five variables on the binary variable “CF+ ” or “CF-.” This figure shows the “mean decrease accuracy” value for each variable. This measure indicates the importance of each variable in the model. A high “mean decrease accuracy” value for a given variable suggests that the variable is important for the model, as the model relies heavily on it to make accurate predictions. On the other hand, if the “mean decrease accuracy” value is low, it indicates that the variable is less important for the model. As shown in this figure, the morphosyntax and working memory have little to no impact on the classification of a child in the CF- or CF+ groups, as opposed to the three parameters related to Cued French use.

These results confirm that the distinction between the children in the CF+ and CF- group is indeed explained by their exposure to Cued French, rather than by language skills of verbal working memory.

## 4. Discussion

### 4.1. Performance of children with CI depends on the speech rehabilitation method

This study was designed to assess the speech perception skills of deaf children who had CI compared to TH children, according to the language rehabilitation approach used (AVT or CF) and to CF reading skills in those using CF (CF+ and CF-). The performance of three groups of children with CI (AVT, CF+, CF-) to a lexicality judgment task was therefore compared with that of children with typical hearing (TH). Results show that the children with CI from the CF- and CF+ have significantly lower performance than children with TH (resp. *p* < 0.001 and *p* = 0.033), and that AVT children also tend to have lower scores than TH children (*p* = 0.07). More precisely, the scores of children with lower CF reading skills (CF-) are considerably more distant from those of children with TH (distance to lower bound of TH confidence interval = 0.75) than the scores of children with high CF reading skills (CF+, distance = 0.25). The children from the AVT group are closer to the TH children (distance = 0.14) than the other two CI groups. Apart from phoneme insertion which was poorly identified in all children and from syllable deletion, which was relatively well identified, the lower scores in the CF- group concerned all phonological alterations.

These results reveal several important points. First, children with cochlear implants who do not consistently use a communication tool have significantly weaker lexicality judgment skills than their peers: the scores of the CF- children are three times more distant from those of TH children than those of the AVT or CF+ children. The lexicality judgment task consisted in detecting phonological distortions in a familiar word, and CF- children seemed to be much less confident about what they can identify as a correct vs. incorrect production. This shows that the cochlear implant alone is not sufficient to develop adequate speech perception skills and that it is important to implement speech and language rehabilitation adapted to the child and the family.

Moreover, children with CI and high cue reading skills have improved speech perception performance compared to their peers with low cue reading skills. Indeed, the analysis shows that cued speech exposure enhances phonological awareness and speech perception even when manual gestures are absent (the stimuli in this study were audiovisual clips, without any cued speech gestures). The scientific literature has amply shown that cued speech reading skills allows for better speech perception when speech is presented with cued speech gestures (Nicholls and Ling, 1982; Périer et al., 1990; Alegria et al., 1999). However, a few studies have suggested that the benefits of cued speech reading skills extend to the perception of speech without any manual gestures, i.e., in visual only, audio-visual, or audio only modality (Kos et al., 2009; LaSasso et al., 2010). This study provides additional knowledge about speech perception without cued speech gestures, because despite the absence of manual cues, CF+ children were able to have adequate speech perception. Manual information from cued speech seems to allow for better encoding of phonological information, which ultimately results in better audiovisual speech processing.

We also found that the intensity of Cued French exposure plays a major role in Cued French reading skills and therefore in speech perception. Indeed, the explanatory variable that most impacts the probability of belonging to the CF+ or CF- group is the frequency of Cued French use. It is important for children to be exposed to Cued French in an intensive and daily way in order to develop adequate Cued French reading skills and improve their speech perception skills. Weekly or occasional exposure is not enough for children to fully benefit from Cued French. Place of use and earliness of exposure are also important factors that can influence Cued French reading skills. Consistent and daily exposure to Cued French at home and at school, starting from birth, can help children to develop better speech perception skills. These data are consistent with previous scientific studies on cued speech (Périer et al., 1990; Charlier and Leybaert, 2000; Leybaert and LaSasso, 2010).

Another finding in this study is that an auditory-focused method, such as AVT, that emphasizes auditory skills, can be effective in children with cochlear implants for the development of age-appropriate speech perception skills. These results are in agreement with some studies on the contribution of AVT to speech perception (Dettman et al., 2011; Percy-Smith et al., 2018; Binos et al., 2021). These findings raise a significant question for both the scientific and therapeutic fields. Indeed, it has been shown in a number of studies that speech perception is multisensory process involving both visual and auditory information. Visual cues significantly improve speech perception, both when the auditory speech is degraded (Sumby and Pollack, 1954) and when the auditory speech is clear (Reisberg et al., 1987). Our results show that, although dissimilar to this multisensory view of the speech perception process, a method that reinforces auditory skills specifically allows deaf children with CI to achieve age-appropriate speech perception performance.

Lastly, the analyses of the β score (which measures the bias of the participants) show that all of the participants in the study were biased toward being more conservative in their responses, and that this bias is present in both children with CI and TH children. This means that the children were more likely to accept an incorrectly pronounced item than to reject a correctly pronounced item, even though the items in the task were high-frequency words and had been presented in a previous picture naming task. Inhibition skills, which involve the ability to control and suppress inadequate behaviors or responses, could be an important part of the process of rejecting an incorrect item, and may not be mature enough in children of that age. An alternative interpretation is that children are used to receiving accurate linguistic input from adults and may therefore be more accepting of slightly incorrect word pronunciations. It is interesting to note that all children in the four groups appeared to use the same strategy and were equally biased in their responses.

### 4.2. Clinical applications

One of the important outcomes of our study is to emphasize the fact that cochlear implantation alone is not sufficient to develop adequate speech perception, as the scores of the CF- group are consistently below those of children with TH and below the scores of the children with high CF proficiency or of those who enrolled in AVT. The second outcome is that Cued French improves speech perception when used intensively and from the early years, since the children in the CF+ group, i.e., children with the highest Cued French proficiency, are precisely those who have benefited from early and intensive exposure. In addition, our results show that AVT also improves perception and may be considered an appropriate option for children with CI. One conclusion that can be drawn from our results is that parental involvement in their child’s rehabilitation is crucial to their speech development, since the two methods we describe rely heavily on the parents’ implication. In order to promote this involvement, the clinician should establish a trusting relationship with the parent and provide support, regardless of the language rehabilitation approach chosen. Another observation concerns education. Our results provide information that may help teachers to better understand the challenges of deafness and language acquisition. Furthermore, our findings on the benefit of the frequency of exposure suggest that if parents use Cued French at home, it should also be implemented at school to increase exposure.

### 4.3. Limitations of the study

The main limitation of this study is the challenge in recruiting participants. Indeed, the sample size in the CI group is small, which reduces the number of children in each subgroup. Moreover, the group of children with CI is inevitably heterogeneous in many aspects such as age at diagnosis, age at intervention and etiology.

There are other uncontrolled factors that may have played a role in speech outcomes for these children. Indeed, even if the age of cochlear implantation did not significantly impact the *d’* score and there was no difference between our groups regarding the age of CI, deaf children still had different mean ages at first CI: children in the AVT group were, on average, 8 months younger than children in the CF+ group and 9 months younger than children in the CF- group when they received their first cochlear implant. Numerous studies have shown that early cochlear implantation, during the critical period of brain neuroplasticity, leads to better speech perception skills (Kral et al., 2016). The variability in age of implantation among children can be explained by the first two principles of AVT, which emphasize the importance of early intervention and the use of advanced hearing aids (see Supplementary Appendix 2). The first principle of AVT prioritizes immediate audiologic management for children, while the second principle ensures that children receive appropriate technology to maximize the benefits of auditory stimulation. These factors could specifically lead parents of early-implanted children to choose AVT. In addition, the duration of CI use and whether the implant is bilateral or unilateral are also important factors to consider in our conclusions. Moreover, parental socio-economic status and parental involvement are variables that could influence the results. It has been shown that parental involvement is a key variable in the speech and language development of the deaf child. In order to more accurately assess the impact of different language rehabilitation approach on speech perception in children with cochlear implants, further exhaustive studies, with larger sample sizes, are needed. These studies should take into account all of the factors that can influence a child’s speech outcomes. These future studies will facilitate discussions about the benefits of various communication approaches and help families make that important decision about which communication method to use with their deaf child.

Finally, one limitation of this study is that the task took place in a quiet room, where listening conditions were at their best. This may not accurately represent the listening conditions that children face in real-conditions classrooms, where there is often background noise and other distractions. This means that the results of the study may not be fully applicable to children’s everyday listening experiences. It would be interesting to conduct further research to evaluate the effects of cued speech and AVT in a more realistic listening environment, such as a typical classroom and noisy environment, to better understand to what extent they can help children improve their speech perception.

## 5. Conclusion

In conclusion, the results of this study show that a multisensory or an auditory-focused speech rehabilitation approach can improve the speech perception performance of deaf children with CI. Indeed, the children enrolled in an AVT program and the children with a good level of cue reading (CF+) have significantly better performance than children who do not consistently use a communication mode (CF-), and their scores are almost similar to those of TH children. Both approaches can be used by parents and professionals to help children develop adequate perceptual skills. Finally, our results confirm that cochlear implantation alone is not sufficient for a child to develop adequate speech perception skills. It is important for careers of children with cochlear implants, such as parents, speech and language therapists, and audiologists, to understand the limitations of speech perception through the cochlear implant and to seek additional support through a specific speech rehabilitation approach, particularly during the early years. It is crucial to provide parents with all available communication options as early as possible.

## Data availability statement

The raw data supporting the conclusions of this article will be made available by the authors, without undue reservation.

## Ethics statement

The studies involving human participants were reviewed and approved by the CERGA, Comité d’Éthique pour les Recherches de Grenoble Alpes-Avis-2022–23. Written informed consent to participate in this study was provided by the participants’ legal guardian/next of kin.

## Author contributions

AV, HL, EG-P, LM, AM, and GM designed the experimental protocol. LV, LM, AV, HL, GM, SA, and CP collected the data. LV, AV, HL, and SG performed data processing and statistical data analysis. LV, HL, and AV wrote the first draft and revised version of the manuscript. All authors contributed to the article and approved the submitted version.

## Consortium EULALIES

Maud Costa (Université Grenoble Alpes, France), Estelle Gillet-Perret (CRTLA, Centre Hospitalier Universitaire Grenoble Alpes, France), Andrea A. N. MacLeod (University of Alberta, Edmonton, Canada), Geneviève Meloni (Université Grenoble Alpes, France and Université de Montréal, Montréal, Canada), Clarisse Puissant (Université Grenoble Alpes, France), and Yvan Rose (Memorial University, Newfoundland, Canada).

## Footnotes

1
https://www.adefav.fr/

2
https://alpc.asso.fr/
