# Supplementary material for: Speech rehabilitation in children with cochlear implants using a multisensory (French Cued Speech) or a hearing-focused (Auditory Verbal Therapy) approach
Source: Front Hum Neurosci. 2023 May 12;17:1152516. doi: 10.3389/fnhum.2023.1152516 (PMC10219235; doi:10.3389/fnhum.2023.1152516)

**Appendix 1**

Cues in Cued French: the five hand positions for vowels (upper left) and the eight handshapes for the consonants (bottom).

Adapted by Laura Machart with permission from the French version of the ALPC

(Association pour la promotion de la Langue française Parlée Complétée: <https://alpc.asso.fr/les-cles-du-code-lpc/>)


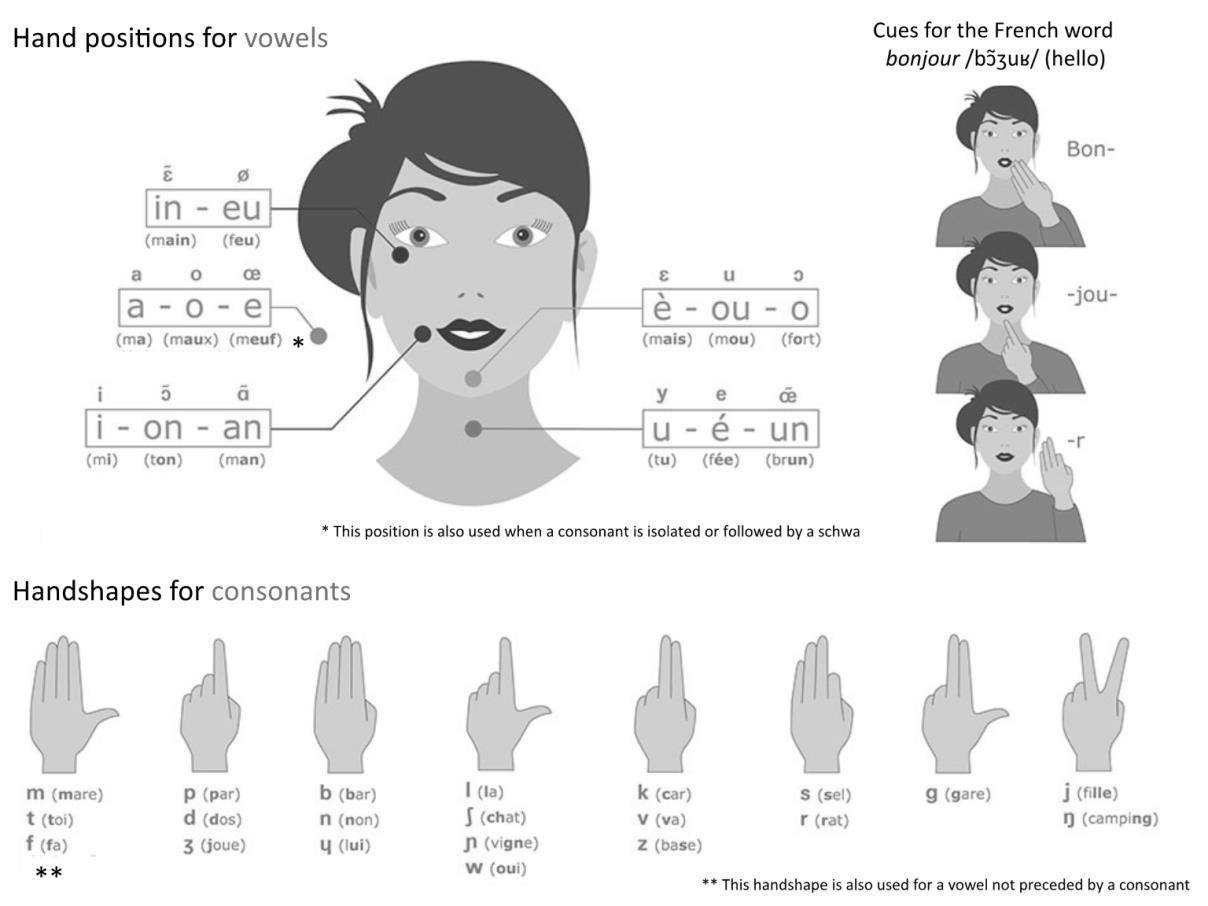

Supplement: Supplementary file 1 [file Data_Sheet_1.docx]
