# Supplementary material for: Speech rehabilitation in children with cochlear implants using a multisensory (French Cued Speech) or a hearing-focused (Auditory Verbal Therapy) approach
Source: Front Hum Neurosci. 2023 May 12;17:1152516. doi: 10.3389/fnhum.2023.1152516 (PMC10219235; doi:10.3389/fnhum.2023.1152516)
Supplement: Supplementary file 2 [file Data_Sheet_2.docx]

**Appendix 2**

Each certified LSL Specialist designation has specific principles professionals must uphold. These principles are the basis of the auditory-verbal practitioner profession.

Principles of Certified LSLS Auditory-Verbal Therapists (LSLS Cert. AVT) from the AG Bell Academy for Listening and Spoken Language (<https://agbellacademy.org/certification/principles-of-lsl-specialists/> )

1. Promote early diagnosis of hearing loss in newborns, infants, toddlers, and young children, followed by immediate audiologic management and auditory-verbal therapy.
2. Recommend immediate assessment and use of appropriate, state-of-the-art hearing technology to obtain maximum benefits of auditory stimulation.
3. Guide and coach parents to help their child use hearing as the primary sensory modality in developing listening and spoken language.
4. Guide and coach parents to become the primary facilitators of their child’s listening and spoken language development through active consistent participation in individualized auditory-verbal therapy.
5. Guide and coach parents to create environments that support listening for the acquisition of spoken language throughout the child’s daily activities.
6. Guide and coach parents to help their child integrate listening and spoken language into all aspects of the child’s life.
7. Guide and coach parents to use natural developmental patterns of audition, speech, language, cognition, and communication.
8. Guide and coach parents to help their child self-monitor spoken language through listening.
9. Administer ongoing formal and informal diagnostic assessments to develop individualized auditory-verbal treatment plans, to monitor progress and to evaluate the effectiveness of the plans for the child and family.
10. Promote education in regular schools with peers who have typical hearing and with appropriate services from early childhood onwards.

**An Auditory-Verbal Practice requires all 10 principles.*

The term “parents” also includes grandparents, relatives, guardians, and any caregivers who interact with the child.
