# Supplementary material for: Speech rehabilitation in children with cochlear implants using a multisensory (French Cued Speech) or a hearing-focused (Auditory Verbal Therapy) approach
Source: Front Hum Neurosci. 2023 May 12;17:1152516. doi: 10.3389/fnhum.2023.1152516 (PMC10219235; doi:10.3389/fnhum.2023.1152516)
Supplement: Supplementary file 3 [file Data_Sheet_3.docx]

**Appendix 3**

Number of items from the lexicality judgement task within each category

| Number of syllables | 1 syllable  2 syllables  3 syllables  4 syllables | N=10  N=18  N=12  N=5 |
| --- | --- | --- |
| Alteration on the segment or structure | Structure  Segment | N=15  N=30 |
| Type of alteration | Consonant  Vowel | N=20  N=10 |
| Position of the alteration | Initial  Medial  Final | N=15  N=14  N=16 |
